# The Book World of Medicine and Science

**Published:** 1900-06-16

**Authors:** 


### The Book World of Medicine and Science.

Diseases of the Gall-Bladder and Bile-Ducts, Including Gall-Stones. By A. W. Mayo Robson, F.R.C.S., Senior Surgeon to the General Infirmary at Leeds, Hunterian Professor of Surgery at the Royal College of Surgeons of England. (London: Baillière, Tindall, and Cox. Pp. 313; 52 Illustrations. Second edition. Price 10s. 6d. net.)

It is satisfactory to see a second edition of a work which was sure to be widely read and thoroughly appreciated. Mr. Mayo Robson has, with the able assistance of Dr. Farquhar Macrae, recast in the present volume his original Hunterian Lectures on the subject of Diseases of the Gall-bladder and Bile-ducts into narrative form, and has added a chapter on gall-stones. He has, moreover, classified his operation cases, appending each series to the particular chapter to which they have reference. The large number of operations which the author has performed since the first appearance of his book has given him further matter from which to draw practical conclusions. He reviews these operations in this edition, and thus increases the value of the treatise. Starting with a short but clear account of the normal anatomy and of the malformations of the gall-bladder and the biliary passages, he proceeds to discuss the inflammatory affections of which they may be the site. These he classifies under the three headings of catarrhal, croupous, and suppurative. It is, perhaps, unfortunate that he employs the term "croupous," which is not so scientific a one as "membranous." This condition of inflammation, leading to the deposit of membrane upon the mucous surface of the gall-bladder and the biliary passage, is one of considerable interest. It closely simulates gall-stones in its symptoms, and unless it rapidly and fully disappears under appropriate treatment of a medical nature, Mr. Robson is convinced that it is well to drain the gall-bladder. In speaking of the suppurative form of the affection, he avers that normal bile is sterile, but that in itself it has little or no antiseptic action. It follows, therefore, that for the presence of pus in the gall-bladder there must have been an infection from without, and he gives a clear and concise account of our present knowledge as to the sources of this infection.

Mr. Robson's is an exhaustive account of the fistulæ that may occur in connection with the gall-bladder and the bile ducts. He draws attention again to the rarity of intestinal obstruction due to the blockage of the intestine by gall-stones. He defines tumours of the gall-bladder as including all the enlargements of that structure, not limiting the term to actual new growths. This is perhaps not altogether wise, for the word "tumour" has at the present day rather a restricted sense, and should possibly be confined in its use to the increase in size caused by the deposition of heterogeneous tissue in an organ. In speaking of cancer of the gall-bladder, Mr. Robson says that it is not uncommon, but is usually secondary. That carcinoma in many cases follows on the irritation produced by gall-stones is true, but this should hardly be spoken of as "secondary," a term which is generally employed to imply a deposit of cancer the source of which is found altogether outside the organ secondarily affected. The new chapter on "Gall-stones" is of high merit, and will well repay a perusal. Towards the close of the book there is an interesting account of duodeno-choledochotomy, or the removal of gall-stones from the common duct by way of the duodenum. The author states that the operation is not so difficult as at first it would appear to be.

The work is well produced, being printed on good paper, which allows of the illustrations being presented with the best effect.

Paralytic Deformities of the Lower Extremities. By E. Noble Smith, F.R.C.S.Edin., Senior Surgeon to the City Orthopædic Hospital, &c. (London: Smith, Elder, and Co. 1900. Pp. 99; 51 Illustrations. Price 5s.)

Possibly there are no other lesions of the lower extremities so distressing as the paralytic deformities which are apt to affect them. Surgical science and mechanical art has done much in the last half-century to relieve the sufferers from these untoward conditions. It is with the object of reviewing the questions which arise as to the most effectual methods of treatment to adopt under the several lesions that present themselves that has led Mr. Noble Smith to publish this short but instructive treatise. There is little or no attempt to deal with the disease or accident that has led to the paralytic state, for treatment of the same is the primary object of the pages. Such treatment is discussed chiefly under two headings—mechanical apparatus and surgical operation. In speaking of the former, Mr. Noble Smith rightly says that it has been in the past too much the custom to depend for treatment of paralytic deformities upon the instrument maker. No doubt the pure mechanicians have shown great ingenuity in providing for the requirements of patients, but it should be the province of the orthopædic surgeon to study the lesions from a scientific point of view so as to act as it were in the capacity of the architect, while the mechanician takes the duty of the builder. In dealing with the surgery of the partial paralyses of the lower limbs, the author gives an account of the various accidents that may follow the simple operation of tenotomy, and insists that want of success is more due to lack of proper after-treatment than to the operation itself. The paragraph on the transplantation of tendons is much too meagre to be of much value, and there is an apparent lack of personal experience in this branch of the subject, although one case that is given as an instance of this line of treatment appears to have been of a most satisfactory character. It is questionable whether the operation of the removal of a wedge-shaped portion of the bones of the tarsus should be classed under the somewhat simple term of osteotomy. Other subjects that are dealt with are the deformities resulting from such lesions as paralysis consequent upon caries of the spine, cerebral palsies in childhood, acute myelitis, lateral sclerosis, and ataxia. The conditions dependent upon anterior poliomyelitis are described, and a series of interesting cases are given, with, in many instances, excellent photographs and drawings. After reading the book through, the impression that is left is that though it contains much useful information, this is not arranged in a very systematic manner, and that much is therefore apt to be lost.

